# Development of the Cerebral Cortex across Adolescence: A Multisample Study of Inter-Related Longitudinal Changes in Cortical Volume, Surface Area, and Thickness

**DOI:** 10.1523/JNEUROSCI.3302-16.2017

**Published:** 2017-03-22

**Authors:** Christian K. Tamnes, Megan M. Herting, Anne-Lise Goddings, Rosa Meuwese, Sarah-Jayne Blakemore, Ronald E. Dahl, Berna Güroğlu, Armin Raznahan, Elizabeth R. Sowell, Eveline A. Crone, Kathryn L. Mills

**Affiliations:** ^1^Department of Psychology, University of Oslo, 0317 Oslo, Norway,; ^2^Department of Preventive Medicine, Keck School of Medicine, University of Southern California, Los Angeles, California 90032,; ^3^Institute of Child Health and; ^4^Institute of Cognitive Neuroscience, University College London, London WC1N 1EH United Kingdom,; ^5^Brain and Development Research Center, Institute of Psychology, and; ^6^Leiden Institute for Brain and Cognition, Leiden University, 2300 RA Leiden, The Netherlands,; ^7^Institute of Human Development, University of California Berkeley, Berkeley, California 94720,; ^8^Child Psychiatry Branch, National Institute of Mental Health, Bethesda, Maryland 20814,; ^9^Children's Hospital of Los Angeles, Los Angeles, California 90027, and; ^10^Department of Psychology, University of Oregon, Eugene, Oregon 97403

**Keywords:** brain development, gray matter, morphometry, MRI, replication

## Abstract

Before we can assess and interpret how developmental changes in human brain structure relate to cognition, affect, and motivation, and how these processes are perturbed in clinical or at-risk populations, we must first precisely understand typical brain development and how changes in different structural components relate to each other. We conducted a multisample magnetic resonance imaging study to investigate the development of cortical volume, surface area, and thickness, as well as their inter-relationships, from late childhood to early adulthood (7–29 years) using four separate longitudinal samples including 388 participants and 854 total scans. These independent datasets were processed and quality-controlled using the same methods, but analyzed separately to study the replicability of the results across sample and image-acquisition characteristics. The results consistently showed widespread and regionally variable nonlinear decreases in cortical volume and thickness and comparably smaller steady decreases in surface area. Further, the dominant contributor to cortical volume reductions during adolescence was thinning. Finally, complex regional and topological patterns of associations between changes in surface area and thickness were observed. Positive relationships were seen in sulcal regions in prefrontal and temporal cortices, while negative relationships were seen mainly in gyral regions in more posterior cortices. Collectively, these results help resolve previous inconsistencies regarding the structural development of the cerebral cortex from childhood to adulthood, and provide novel insight into how changes in the different dimensions of the cortex in this period of life are inter-related.

**SIGNIFICANCE STATEMENT** Different measures of brain anatomy develop differently across adolescence. Their precise trajectories and how they relate to each other throughout development are important to know if we are to fully understand both typical development and disorders involving aberrant brain development. However, our understanding of such trajectories and relationships is still incomplete. To provide accurate characterizations of how different measures of cortical structure develop, we performed an MRI investigation across four independent datasets. The most profound anatomical change in the cortex during adolescence was thinning, with the largest decreases observed in the parietal lobe. There were complex regional patterns of associations between changes in surface area and thickness, with positive relationships seen in sulcal regions in prefrontal and temporal cortices, and negative relationships seen mainly in gyral regions in more posterior cortices.

## Introduction

Insight into postnatal human brain development has been greatly enhanced over the last two decades by the use of imaging methods, particularly magnetic resonance imaging (MRI; Jernigan et al., 2011; Blakemore, 2012; Giedd et al., 2015). There are, however, still fundamental disagreements across available studies regarding the developmental patterns and precise trajectories for cortical volume and its distinct components, surface area and thickness (Mills and Tamnes, 2014). To try to resolve the inconsistencies and provide clues about the processes driving the changes in the different dimensions of the cerebral cortex from childhood to adulthood, we investigated the development of cortical structure concurrently in four separate longitudinal samples, and directly assessed how changes in different cortical measures are inter-related.

Previous results are particularly contradictory with regard to the development of cortical thickness, with some studies reporting increases until late childhood, while others finding continuous thinning from early or midchildhood (Walhovd et al., 2016). Inconsistencies across studies of development of cortical structure may have resulted from varying sample characteristics, image acquisition, image processing, including quality-control (QC) procedures and software used, and/or statistical analyses and curve fitting (Fjell et al., 2010; Sullivan et al., 2011; Aubert-Broche et al., 2013; Ducharme et al., 2016; Mills et al., 2016). One approach to try to clarify these inconsistencies is to conduct multisample studies following current standards and recommendations for processing and analysis. Here, we build upon a recent such study in which we reported replicable models for gross structural brain development between childhood and adulthood (Mills et al., 2016).

Cortical volume is determined by surface area and thickness, and these components are influenced by different evolutionary (Geschwind and Rakic, 2013), genetic (Chen et al., 2013; Kremen et al., 2013), and cellular (Chenn and Walsh, 2002) processes, and show unique changes across different stages of life (Brown et al., 2012; Storsve et al., 2014; Wierenga et al., 2014; Lyall et al., 2015; Amlien et al., 2016). Knowledge about the relative contributions of surface area and thickness to developmental cortical volume changes, and the relationship between changes in surface area and thickness during adolescence, may provide important, although indirect, clues for understanding the biological processes underlying development of cortical structure. In prenatal and perinatal life, the primary processes driving surface-area expansion and thickening are cortical column generation and genesis of neurons within columns, respectively (Rakic, 1988; Bhardwaj et al., 2006). The processes underlying changes in cortical structure throughout childhood and adolescence are less well understood, although we know that the protracted human brain development involves increasing caliber and myelination of axons (Yakovlev and Lecours, 1967; Benes, 1989; Benes et al., 1994), and that early synaptogenesis is followed by pruning (Huttenlocher and Dabholkar, 1997; Petanjek et al., 2011).

To increase our confidence in current interpretations about how the cerebral cortex grows and to gain knowledge that might help us understand the processes driving its development, the present study aimed to (1) characterize the regional developmental trajectories of cortical volume, surface area, and thickness across adolescence in four separate longitudinal samples and (2) directly test how changes in the distinct cortical components are inter-related. Each independent dataset was analyzed separately to examine the consistency and replicability of the results across sample and image-acquisition specifics.

## Materials and Methods

### 

#### 

##### Participants.

This study used four separate datasets: Child Psychiatry Branch (CPB), Pittsburgh (PIT), Neurocognitive Development (NCD), and Braintime (BT). Each of these included data about typically developing participants collected at four separate sites (National Institutes of Health, University of Pittsburgh, University of Oslo, Leiden University) in three countries (United States, Norway, Netherlands). All datasets were collected using accelerated longitudinal designs. Each separate study was approved by a local review board. For the CPB dataset, participants and scans were selected from a pool of >1000 scans for their quality and number of time points per individual. For the PIT, NCD, and BT datasets, respectively, 126, 111, and 299 participants were recruited and scanned at baseline. Of these, 20, 26, and 45 dropped out at follow up, and an additional 33, 9, and 45 were excluded based on the QC of the MRI data (see below). The final CPB, PIT, NCD, and BT datasets included 30, 73, 76, and 209 participants, respectively. In total, the present study includes 388 participants (199 females) and 854 scans covering the age range of 7–29 years old (Table 1). Details regarding participant recruitment have been described previously for each sample separately (Tamnes et al., 2013; Herting et al., 2014; Mills et al., 2014b; Braams et al., 2015) and together (Mills et al., 2016).

**Table 1.** Participant demographics and MRI acquisition parameters for each sample

	CPB	PIT	NCD	BT
*N* participants (females)	30 (9)	73 (41)	76 (37)	209 (112)
Age mean (SD)*^a^*	15.6 (1.7) years	13.4 (0.9) years	15.2 (3.3) years	15.7 (3.6) years
Age range	7.0–29.9 years	10.1–16.2 years	8.2–21.9 years	8.0–26.6 years
*N* scans (individual range)	138 (3–6)	146 (2)	152 (2)	418 (2)
Scan interval mean (SD)	3.7 (2.2) years	2.2 (0.4) years	2.6 (0.2) years	2.0 (0.1) years
Scan interval range	1.1–14.0 years	1.5–3.7 years	2.4–3.2 years	1.6–2.5 years
Scanner	GE Signa 1.5 T	Siemens Allegra 3 T	Siemens Avanto 1.5 T	Philips Achieva 3 T
Repetition time (TR)	2400 ms	1540 ms	2400 ms	9.76 ms
Echo time (TE)	5.00 ms	3.04 ms	3.61 ms	4.59 ms
Voxel size	0.938 × 0.938 × 1.5 mm	1.0 × 1.0 × 1.0 mm	1.25 × 1.25 × 1.20 mm	0.875 × 0.875 × 1.2 mm

*^a^*Mean across available time points.

##### Image acquisition and processing.

T1-weighted anatomical scans were obtained at four different sites using different scanners and sequences. Only key parameters are summarized here (Table 1), as details regarding image acquisition at each site were described in detail previously (Mills et al., 2016). At each site, a radiologist reviewed all scans for gross abnormalities. Image processing was performed with FreeSurfer 5.3 (RRID:SCR_001847), which is documented and freely available online (http://surfer.nmr.mgh.harvard.edu/). The technical details of the procedures were described in detail previously (Dale et al., 1999; Fischl et al., 1999, 2002). The processing stream includes motion correction (Reuter et al., 2010), removal of nonbrain tissue using a hybrid watershed/surface-deformation procedure (Ségonne et al., 2004), automated Talairach transformation, nonparametric nonuniform intensity normalization (Sled et al., 1998), tessellation of the gray–white matter boundary, automated topology correction (Fischl et al., 2001; Ségonne et al., 2007), and surface deformation following intensity gradients to optimally place the gray–white and gray–CSF borders at the location where the greatest shift in intensity defines the transition to the other tissue class (Dale and Sereno, 1993; Dale et al., 1999; Fischl and Dale, 2000). Each cortical model was registered to a spherical atlas using individual cortical folding patterns to match cortical geometry across participants (Dale et al., 1999).

Images were then processed using FreeSurfer 5.3's longitudinal stream (Reuter et al., 2012). This process includes the creation of an unbiased within-subject template space and image using robust, inverse consistent registration (Reuter et al., 2010). Several processing steps, such as skull stripping, Talairach transforms, and atlas registration, as well as spherical surface maps and parcellations, were then initialized with common information from the within-subject template, significantly increasing reliability and statistical power (Reuter et al., 2012). The QC procedure was coordinated across sites so that all images were visually inspected post-processing by trained operators for accuracy, but no editing was performed.

Surface maps for cortical volume, surface area (white surface), and thickness, as well as symmetrized annual percentage change (APC; i.e., the linear annual rate of change with respect to the average volume/area/thickness measure across all available time points) over all available observations for each measure, were generated and smoothed with a Gaussian kernel of full-width at half-maximum of 15 mm. Additionally, we computed global total cortical volume, total surface area, and weighted mean thickness (with each label contributing to the mean according to its area) for each time point for each subject across all labels in the Desikan–Killiany cortical parcellation (Desikan et al., 2006), and APC for each of the measures. Similar variables were calculated for the frontal (including anterior cingulate), temporal (including insula), parietal (including posterior and retrosplenial cingulate), and occipital lobes, and for each label across both hemispheres.

##### Statistical analysis.

First, spaghetti plots and longitudinal curve fitting were performed using the Multimodal Imaging Laboratory data portal (Bartsch et al., 2014; Vidal-Pineiro et al., 2016), which uses functions freely available through the statistical environment R (http://www.r-project.org/, RRID:SCR_001905). Cortical volume, surface area, and thickness measures from each time point, adjusted for the effect of sex, were introduced as predicted variables in generalized additive mixed model (GAMM) analyses where the predictor was age, with *k* parameters specifying the stiffness of the model curves set to five (except for temporal lobe volume where four was used so the models would converge). The main effect of sex was adjusted for through linear mixed-effect models. GAMM can be represented as the following formula: *G*(*y*) = X*α + ‖_*j*=1_^*p*^(*x_j_*) + *f_j_*(*x_j_*) + *Zb* + ε, where G(·) is a monotonic differentiable link function, α is the vector of regression coefficients for the fixed parameters; X* is the fixed-effects matrix; *f_j_* is the smooth function of the covariate *x_j_*; *Z* is the random-effects model matrix; *b* is the vector of random-effects coefficients; and ε is the residual error vector (Wood, 2006). GAMM fitting was visualized over its correspondent spaghetti plots. Estimated mean values across sex were used as display values. GAMM provides accurate delineations of developmental trajectories, as it avoids some of the inherent weaknesses of global polynomial models, e.g., quadratic and cubic models, where the timing of peaks and the end points of the trajectories may be substantially affected by irrelevant factors, such as the age range sampled (Fjell et al., 2010).

Second, as background analyses before testing for inter-relationships between cortical volume, surface area, and thickness, mean global and lobar APC values for each cortical measure for each sample were calculated, one-sample *t* tests were used to test whether the APC values were significantly different from zero, and ANOVAs with Tukey's HSD *post hoc* comparisons were performed to test for sample differences. For each sample, we then performed vertex-wise general linear models (GLMs) as implemented in FreeSurfer 5.3, testing whether APCs for each of the measures were significantly different from zero, with sex, age (mean across time points), and their interaction as covariates.

Third, regional relationships between changes in cortical volume, surface area, and thickness were initially tested for by means of partial correlations between global and lobar APCs in each measure, with sex and mean age as covariates. Then, a series of GLMs were performed in FreeSurfer to test for vertex-wise change–change relationships among the different measures across the cortical surface. APC maps for each measure were entered as per-vertex regressors of interest to the other measures, with sex, mean age, and their interaction as covariates.

All regional (global and lobar) results were Bonferroni-corrected by a factor of five (reflecting the number of regions), corresponding to a corrected α of *p* < 0.01. For all vertex-wise analyses, the data were tested against an empirical null distribution of maximum cluster size across 10,000 iterations using Z Monte Carlo simulations as implemented in FreeSurfer (Hayasaka and Nichols, 2003; Hagler et al., 2006) synthesized with a cluster-forming threshold of *p* < 0.05 (two-sided), yielding clusters fully corrected for multiple comparisons across the surfaces. Cluster-wise corrected *p* < 0.05 was regarded to be significant.

## Results

### Delineating cortical developmental trajectories

**Figure 1.**
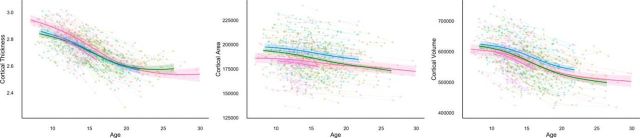
Developmental trajectories for global cortical measures. Spaghetti plots of mean cortical thickness, total cortical surface area, and total cortical volume, controlling for sex. The colored lines represent the GAMM fitting while the lighter colored areas correspond to the 95% confidence intervals. Pink, CPB; purple, PIT; blue, NCD; green, BT.

**Figure 2.**
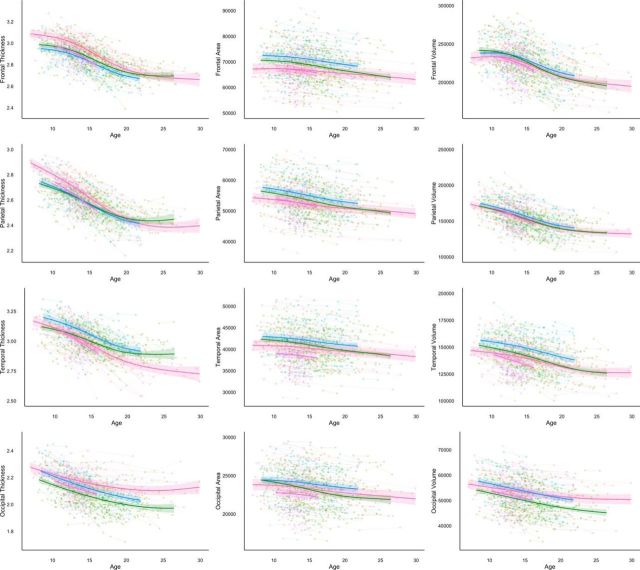
Developmental trajectories for lobar cortical measures. Spaghetti plots of lobar cortical thickness, surface area, and volume, controlling for sex. The colored lines represent the GAMM fitting while the lighter colored areas correspond to the 95% confidence intervals. Pink, CPB; Purple, PIT; blue, NCD; green, BT.

To accurately characterize longitudinal developmental trajectories, global (Fig. 1) and lobar (Fig. 2) cortical volume, surface area, and thickness measures were visualized as spaghetti plots fitted with GAMM. Total cortical volume decreased across the whole age range in all four samples, with slightly accelerated decreases in the adolescent period compared with late childhood and early adulthood. Total cortical surface area showed nearly linear decreases in all four samples, but appeared overall greater for the two European samples (NCD, BT) and had a somewhat flatter slope for one of the US samples (CPB). Mean cortical thickness showed highly overlapping nonlinear trajectories, with accelerated thinning in adolescence.

The lobar trajectories were overall similar to the global results, although some regional differences were also evident. For example, cortical volume showed a relatively stable trajectory in late childhood in the frontal lobe, and the accelerated thinning in adolescence was most clearly seen in the frontal lobe, while decelerating trajectories with increasing age were seen for thickness in the parietal and occipital lobes.

### Mapping longitudinal cortical change

On average for each sample, and on both the global and lobar level, cortical volume, thickness, and surface area all showed negative change rates, i.e., reductions with increasing age (Table 2; see Table 3 for APCs for all measures for all labels in the cortical parcellation). For the global measures and within all four samples, cortical volume showed the largest decrease (APCs from −1.10 to −1.87; sample mean, −1.43), followed by thickness (APCs from −0.83 to −1.38; sample mean, −1.03), and finally surface area (APCs from −0.36 to −0.71; sample mean, −0.55). Although the ranking of APCs in the different global measures was the same in all four samples, there were also significant sample differences in all three measures (Table 2).

**Table 2.** Global and lobar change in cortical volume, surface area, and thickness for each sample

	CPB	PIT	NCD	BT	Significant sample differences
Cortical volume	−1.10	−1.87	−1.15	−1.60	CPB–PIT, PIT–NCD, NCD–BT
Frontal lobe	−1.04	−1.67	−1.08	−1.60	
Temporal lobe	−0.88	−1.65	−0.99	−1.32	CPB–PIT, PIT–NCD
Parietal lobe	−1.54	−2.53	−1.49	−1.98	CPB–PIT, PIT–NCD, PIT–BT, NCD–BT
Occipital lobe	−0.61	−1.31	−0.84	−1.28	CPB–PIT, CPB–BT, NCD–BT
Cortical surface area	−0.36	−0.61	−0.53	−0.71	CPB–PIT, CPB–BT, NCD–BT
Frontal lobe	−0.29	−0.48	−0.47	−0.61	CPB–BT
Temporal lobe	−0.31	−0.52	−0.43	−0.59	CPB–BT
Parietal lobe	−0.50	−0.90	−0.71	−0.87	CPB–PIT, CPB–BT
Occipital lobe	−0.34	−0.46	−0.47	−0.85	CPB–BT, PIT–BT NCD–BT
Cortical thickness	−0.93	−1.38	−0.83	−0.98	PIT–NCD, PIT–BT
Frontal lobe	−0.93	−1.29	−0.83	−0.99	
Temporal lobe	−0.89	−1.43	−0.82	−0.90	PIT–NCD, PIT–BT
Parietal lobe	−1.15	−1.63	−0.94	−1.17	PIT–NCD, PIT–BT
Occipital lobe	−0.45	−0.90	−0.58	−0.66	

Values displayed are mean symmetrized APC for each measure. All APC values were significantly different from zero (*p* < 0.001). Differences among the samples were tested with ANOVAs (*p* < 0.05, Bonferroni-corrected, factor of 5) with Tukey's HSD *post hoc* comparisons (*p* < 0.05) and those showing significant differences are listed in the far right column.

**Table 3.** Regional change in cortical volume, surface area and thickness for each sample

	Cortical volume				Cortical surface area				Cortical thickness			
	CPB	PIT	NCD	BT	CPB	PIT	NCD	BT	CPB	PIT	NCD	BT
Frontal, superior	−1.04	−1.63	−0.99	−1.66	−0.21	−0.33	−0.36	−0.50	−0.92	−1.36	−0.80	−1.09
Frontal, rostral middle	−1.27	−2.14	−1.42	−2.12	−0.52	−0.74	−0.64	−0.79	−1.25	−1.73	−1.26	−1.34
Frontal, caudal middle	−1.38	−2.09	−1.55	−1.90	−0.54	−0.79	−0.83	−0.80	−0.94	−1.24	−0.87	−1.02
Frontal, lateral orbital	−1.02	−1.78	−1.14	−1.29	−0.14	−0.62	−0.38	−0.34	−0.93	−1.27	−0.78	−0.91
Frontal, pars orbitalis	−1.06	−1.67	−1.12	−1.65	−0.26	−0.45	−0.47	−0.95	−0.93	−1.19	−0.71	−0.87
Frontal, pars triangularis	−0.96	−1.74	−0.99	−1.70	−0.06	−0.26	−0.24	−0.62	−1.02	−1.49	−0.96	−1.15
Frontal, pars opercularis	−1.02	−1.66	−1.15	−1.51	−0.28	−0.46	−0.47	−0.50	−0.95	−1.28	−0.86	−1.00
Frontal, precentral	−0.55	−1.24	−0.57	−1.07	−0.20	−0.36	−0.42	−0.50	−0.41	−0.78	−0.28	−0.55
Frontal, pole	−0.42	−0.95	0.17	−2.38	−0.32	−0.58	0.31	−1.52	−0.54	−0.77	−0.45	−0.89
Frontal, medial orbital	−1.12	−1.26	−1.46	−1.33	−0.18	−0.29	−0.55	−0.62	−1.08	−1.01	−1.06	−0.79
Frontal, rostral anterior cingulate	−1.07	−0.40	−0.44	−0.82	−0.22	−0.02	−0.15	−0.40	−1.14	−1.08	−0.68	−0.49
Frontal, caudal anterior cingulate	−1.11	−1.06	−1.08	−1.26	−0.29	−0.08	−0.26	−0.31	−1.05	−1.08	−1.03	−1.05
Frontal, paracentral	−1.33	−2.15	−1.36	−1.62	−0.41	−0.70	−0.62	−0.92	−1.01	−1.69	−0.95	−1.07
Temporal, superior	−0.73	−1.59	−0.80	−1.28	−0.22	−0.37	−0.26	−0.56	−0.79	−1.35	−0.76	−0.82
Temporal, middle	−0.74	−1.50	−0.92	−1.42	−0.19	−0.34	−0.26	−0.52	−1.07	−1.64	−1.06	−1.06
Temporal, inferior	−0.98	−1.90	−1.31	−1.52	−0.38	−0.66	−0.51	−0.65	−1.02	−1.69	−1.06	−1.01
Temporal, banks superior temporal sulcus	−2.06	−3.12	−1.91	−2.06	−0.76	−1.23	−1.02	−0.84	−1.91	−2.45	−1.32	−1.57
Temporal, transverse	−1.27	−1.91	−1.36	−1.47	−0.45	−1.08	−0.95	−1.21	−0.71	−0.75	−0.51	−0.49
Temporal, pole	0.28	−0.85	0.59	0.01	0.02	−0.28	0.27	−0.07	0.14	−0.66	0.13	0.09
Temporal, entorhinal	−0.43	−0.45	−0.56	−0.25	−0.36	−0.22	−0.40	−0.34	0.07	−0.13	0.08	0.11
Temporal, parahippocampal	−1.32	−1.77	−0.83	−1.20	−0.29	−0.51	−0.27	−0.52	−1.03	−1.49	−0.62	−0.81
Temporal, fusiform	−1.28	−2.02	−1.19	−1.50	−0.23	−0.43	−0.38	−0.64	−1.04	−1.53	−0.82	−0.95
Temporal, insula	−0.58	−0.94	−0.85	−1.12	−0.46	−0.62	−0.76	−0.54	−0.54	−0.95	−0.53	−0.92
Parietal, superior	−1.64	−2.58	−1.45	−2.02	−0.55	−0.83	−0.80	−0.93	−1.18	−1.66	−0.83	−1.08
Parietal, inferior	−1.65	−2.58	−1.67	−2.15	−0.53	−1.01	−0.82	−0.88	−1.34	−1.72	−1.05	−1.32
Parietal, supramarginal	−1.48	−2.71	−1.53	−2.01	−0.51	−1.01	−0.71	−0.86	−1.07	−1.64	−0.93	−1.14
Parietal, postcentral	−1.23	−2.26	−1.27	−1.64	−0.42	−0.73	−0.57	−0.96	−0.77	−1.22	−0.74	−0.74
Parietal, precuneus	−1.60	−2.65	−1.44	−1.99	−0.46	−0.90	−0.64	−0.78	−1.26	−1.92	−0.99	−1.42
Parietal, posterior cingulate	−1.52	−2.17	−1.45	−1.78	−0.57	−0.90	−0.57	−0.59	−1.18	−1.54	−1.05	−1.34
Parietal, retrosplenial cingulate	−1.46	−2.37	−1.64	−2.08	−0.44	−0.98	−0.54	−0.73	−1.07	−1.45	−1.16	−1.41
Occipital, lateral	−0.48	−1.38	−0.97	−1.43	−0.55	−0.36	−0.51	−1.03	−0.30	−1.12	−0.81	−0.72
Occipital, cuneus	−0.94	−1.36	−0.90	−1.36	−0.37	−0.58	−0.54	−0.80	−0.72	−0.94	−0.53	−0.84
Occipital, pericalcarine	0.05	0.35	0.37	0.11	0.17	−0.08	−0.23	−0.33	−0.21	0.21	0.25	0.17
Occipital, lingual	−0.89	−1.69	−0.99	−1.39	−0.20	−0.72	−0.48	−0.84	−0.62	−0.91	−0.50	−0.67

Values displayed are mean symmetrized APC for each measure.

For the lobar measures, cortical volume consistently showed the same ranking of APCs within all four samples, with the largest decrease in the parietal lobe, followed by the frontal, the temporal, and finally the occipital lobe. The parietal lobe also showed the largest decrease in both cortical surface area and thickness in all four samples, and the occipital lobe consistently showed the smallest decrease in cortical thickness. Except for frontal lobe volume and thickness and occipital lobe thickness, there were significant sample differences in the lobar APC values in all measures (Table 2).

**Figure 3.**
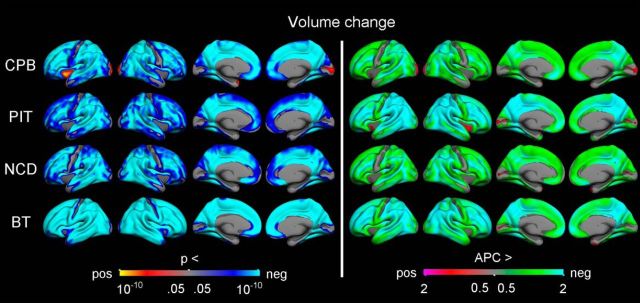
Longitudinal change in cortical volume. GLMs were used to test the statistical significance of APC in volume across the brain surface in each sample, with sex and mean age included as covariates. The results were corrected for multiple comparisons using cluster-size inference. Left, Uncorrected *p* values within the corrected significant clusters. Right, Rates of change.

**Figure 4.**
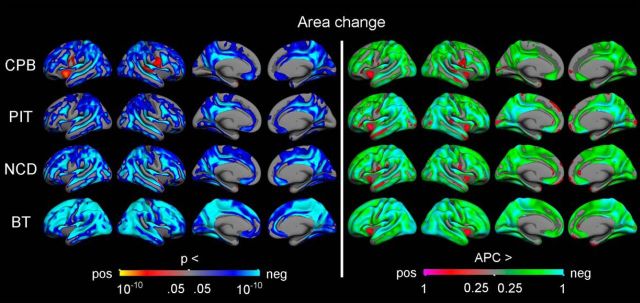
Longitudinal change in cortical surface area. GLMs were used to test the statistical significance of APC in area across the brain surface in each sample, with sex and mean age included as covariates. The results were corrected for multiple comparisons using cluster-size inference. Left, Uncorrected *p* values within the corrected significant clusters. Right, Rates of change.

**Figure 5.**
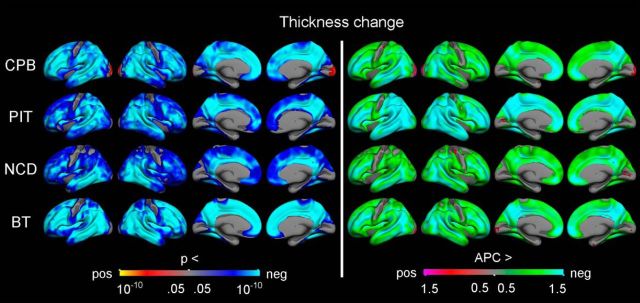
Longitudinal change in cortical thickness. GLMs were used to test the statistical significance of APC in thickness across the brain surface in each sample, with sex and mean age included as covariates. The results were corrected for multiple comparisons using cluster-size inference. Left, Uncorrected *p* values within the corrected significant clusters. Right, Rates of change.

Vertex-wise surface maps were then created to visualize the statistical significance (controlling for sex and mean age) and rate of APCs in cortical volume (Fig. 3), surface area (Fig. 4), and thickness (Fig. 5) for each of the samples separately. Corrected significant negative changes were seen for all three measures for extensive portions of the cerebral cortex in all four samples. Some exceptions or sample differences were noted. For volume, significant increases or no effects were seen around the central sulcus and in insular, medial temporal, and medial occipital cortices. For surface area compared with the other two measures, more regions did not show significant APCs, especially gyral regions in the three smaller samples. And finally, for thickness, the rate decrease in most regions was larger for the sample with a narrower age range in adolescence (PIT), than for the other three samples. Note that the scale for rate of APCs varies across the different measures.

### Testing for inter-related changes in different cortical components

Relationships between global and lobar changes in surface area and volume and in thickness and volume were first tested with partial correlations, controlling for sex and mean age (Table 4). All samples showed large positive associations between thickness APCs and volume APCs (*r* = 0.72–0.95) for both global and all lobar measures. For global measures, the associations between surface area APC and volume APC varied from small to medium (*r* = 0.16–0.55), while for the lobar measures medium-to-large positive associations (*r* = 0.51–0.76) were seen in the frontal lobe in three of the samples (PIT, NCD, BT), in the temporal lobe in two samples (NCD, BT), and in the parietal lobe in one sample (PIT).

**Table 4.** Relationships between changes in surface area and volume and between changes in thickness and volume, for each sample

	CPB	PIT	NCD	BT
Area change−volume change				
Global cortex	0.16 (0.427)	0.47*^a^* (<0.001)	0.55*^a^* (<0.001)	0.51*^a^* (<0.001)
Frontal lobe	0.26 (0.179)	0.68*^a^* (<0.001)	0.69*^a^* (<0.001)	0.51*^a^* (<0.001)
Temporal lobe	0.04 (0.827)	0.37*^a^* (0.002)	0.69*^a^* (<0.001)	0.52*^a^* (<0.001)
Parietal lobe	0.00 (0.985)	0.76*^a^* (<0.001)	0.33*^a^* (0.005)	0.29*^a^* (<0.001)
Occipital lobe	0.37 (0.056)	0.07 (0.543)	0.01 (0.922)	−0.07 (0.304)
Thickness change−volume change				
Global cortex	0.87*^a^* (<0.001)	0.85*^a^* (<0.001)	0.93*^a^* (<0.001)	0.93*^a^* (<0.001)
Frontal lobe	0.86*^a^* (<0.001)	0.87*^a^* (<0.001)	0.94*^a^* (<0.001)	0.93*^a^* (<0.001)
Temporal lobe	0.90*^a^* (<0.001)	0.95*^a^* (<0.001)	0.95*^a^* (<0.001)	0.92*^a^* (<0.001)
Parietal lobe	0.90*^a^* (<0.001)	0.72*^a^* (<0.001)	0.91*^a^* (<0.001)	0.93*^a^* (<0.001)
Occipital lobe	0.82*^a^* (<0.001)	0.79*^a^* (<0.001)	0.94*^a^* (<0.001)	0.89*^a^* (<0.001)

Values displayed are partial correlations between symmetrized APC in different cortical measures, controlling for sex and age, with *p* values in parentheses.

*^a^p* < 0.05 (Bonferroni-corrected, factor of 5).

**Figure 6.**
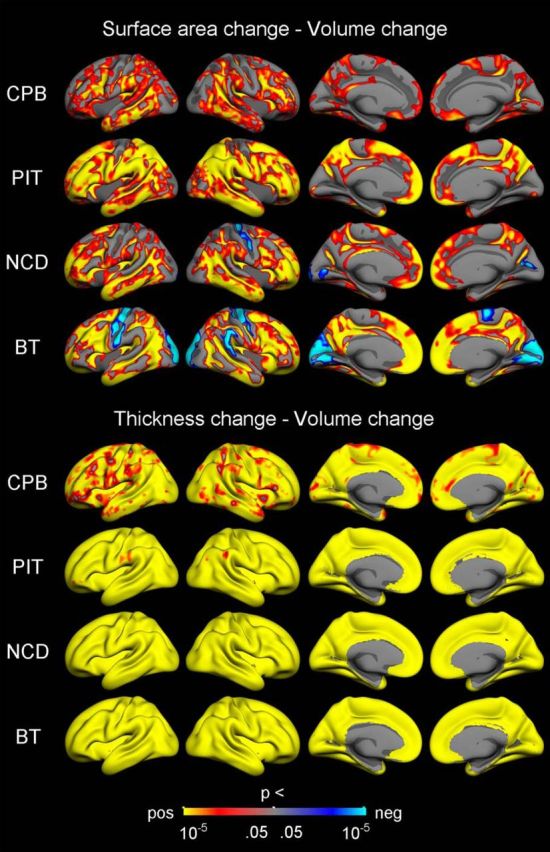
Relationships between change in surface area and thickness and change in volume. Vertex-wise *p* value maps from GLMs testing the relationships between symmetrized APC in different cortical measures, with sex and mean age included as covariates. The results were corrected for multiple comparisons using cluster-size inference. Uncorrected *p* values within the corrected significant clusters are shown. Red–yellow reflects a positive relationship, where a relatively large decrease in one measure is associated with a relatively large decrease in the other measure. Blue–cyan reflects a negative relationship, in which a relatively large decrease on one measure is associated with a relatively small decrease or increase on the other measure.

The results from the per-vertex regression models (controlling for sex and mean age) of surface area APCs and volume APCs and of thickness APCs and volume APCs, respectively, confirmed these general patterns (Fig. 6). Highly significant positive associations between thickness change and volume change were observed across nearly the entire cerebral cortex in all four samples. In comparison, the associations between area change and volume change were not as strong or widespread, and in several regions in two of the samples (NCD, BT) even in the opposite direction (i.e., negative).

Inter-relationships between APCs in cortical surface area and APCs in cortical thickness were first tested on global and lobar measures with partial correlations, controlling for sex and mean age (Table 5). For the global measures, a significant small positive association (*r* = 0.22) was seen in the largest sample (BT). For the lobar measures, significant small-to-medium positive associations (*r* = 0.21–0.52) were seen for the frontal lobe in three samples (PIT, NCD, BT) and for the temporal lobe in two samples (NCD, BT), while significant medium negative associations (*r* = −0.46 to −0.48) were seen for the occipital lobe in two samples (PIT, BT). Inconsistent with the other samples, the CPB sample showed negative, although nonsignificant, associations for the frontal and temporal lobe measures, possibly related to the younger average baseline age of this sample.

**Table 5.** Global and lobar relationships between changes in cortical surface area and thickness for each sample

Surface area change−thickness change	CPB	PIT	NCD	BT
Global cortex	−0.23 (0.231)	0.03 (0.827)	0.30 (0.011)	0.22*^a^* (0.001)
Frontal lobe	−0.13 (0.524)	0.38*^a^* (0.001)	0.49*^a^* (<0.001)	0.21*^a^* (0.002)
Temporal lobe	−0.21 (0.278)	0.15 (0.219)	0.52*^a^* (<0.001)	0.23*^a^* (0.001)
Parietal lobe	−0.32 (0.098)	0.18 (0.143)	−0.01 (0.940)	−0.03 (0.656)
Occipital lobe	−0.18 (0.374)	−0.46*^a^* (<0.001)	−0.28 (0.014)	−0.48*^a^* (<0.001)

Values displayed are partial correlations between symmetrized APC in cortical surface area and thickness, controlling for sex and age, with *p* values in parentheses.

*^a^p* < 0.05 (Bonferroni-corrected, factor of 5).

**Figure 7.**
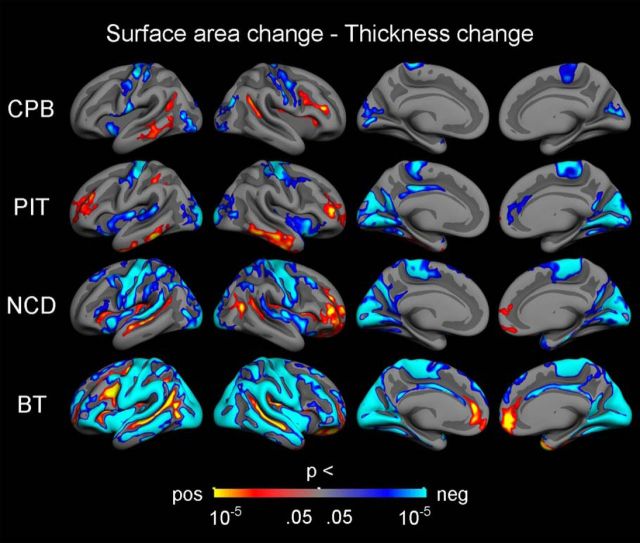
Relationships between change in surface area and change in thickness. Vertex-wise *p* value maps from GLMs testing the relationships between symmetrized APC in different cortical measures, with sex and mean age included as covariates. The results were corrected for multiple comparisons using cluster-size inference. Uncorrected *p* values within the corrected significant clusters are shown. Red–yellow reflects a positive relationship, where a relatively large decrease in one measure is associated with a relatively large decrease in the other measure. Blue–cyan reflects a negative relationship, in which a relatively large decrease on one measure is associated with a relatively small decrease or increase on the other measure.

To investigate these regional differences in more detail, per-vertex regression models (controlling for sex and mean age) of surface area APCs and thickness APCs were performed (Fig. 7). In all four samples, positive associations were observed in lateral prefrontal and temporal cortices, while negative associations were seen around the central sulcus and in paracentral, insular, and both lateral and medial occipital cortices. Generally, the negative associations were more widespread than the positive. Importantly, the vertex-wise results also revealed a complex topographic pattern of positive and negative associations, with positive relations mainly seen in sulcal regions and negative relations seen in gyral and insular regions. The exact location of some of the relations between surface area APCs and thickness APCs did, however, vary across samples, e.g., three samples (CPB, NCD, BT) showed positive associations in the superior temporal sulcus, while one sample (PIT) showed positive associations in the middle and interior temporal cortices. Also, the extent of both the positive and negative associations appeared to be related to sample size, with the spatially most limited effects seen in the CPB sample and the most widespread effects, especially negative associations, seen in the BT sample. In the three smallest samples (CPB, PIT, NCD), most vertices did not show significant associations.

## Discussion

The current multisample neuroimaging study aimed to examine the development of the human cerebral cortex across adolescence in four independent longitudinal samples. The results were generally consistent across samples and showed the following: (1) that the cerebral cortex undergoes widespread and regionally variable nonlinear decreases in volume and thickness with increasing age, and comparatively smaller steady decreases in surface area; (2) that the dominant contributor to cortical volume reductions during adolescence is cortical thinning; and (3) that there are complex regional and topological patterns in the relationships between longitudinal changes in surface area and thickness. Together, the results increase confidence in conclusions about structural cortical development and provide novel insight into how changes in distinct cortical components are linked.

In the first 2 years of life, cortical volume, surface area, and thickness all increase over time (Gilmore et al., 2012; Lyall et al., 2015). There are almost no data for the following years of early childhood due to head motion-related MRI artifacts, and there are inconsistencies across studies regarding developmental patterns and trajectories of different structural measures from midchildhood to adulthood (Mills and Tamnes, 2014; Walhovd et al., 2016). Early longitudinal studies suggested continued increases in cortical volume until late childhood or early adolescence (Giedd et al., 1999; Lenroot et al., 2007; Raznahan et al., 2011), while later longitudinal studies (Lebel and Beaulieu, 2011; Aubert-Broche et al., 2013; Tamnes et al., 2013; Mills et al., 2014a, 2016; Wierenga et al., 2014), as well as the current results, indicate that cortical volume is at its highest earlier in childhood and decreases in late childhood and throughout adolescence.

Previous longitudinal studies are particularly conflicting with regard to cortical thickness, with some indicating inverted-U trajectories from childhood to adulthood, with estimates of peak thickness in late childhood (Shaw et al., 2007, 2008; Raznahan et al., 2011), while others show widespread monotonic decreases during childhood and adolescence (Sowell et al., 2004; Shaw et al., 2006; van Soelen et al., 2012; Mutlu et al., 2013; Alexander-Bloch et al., 2014; Mills et al., 2014a; Wierenga et al., 2014; Zielinski et al., 2014; Fjell et al., 2015; Zhou et al., 2015; Ducharme et al., 2016; Vijayakumar et al., 2016). Our results support the conclusion of decreasing cortical thickness with increasing age during late childhood and across adolescence. Fewer longitudinal studies have investigated cortical surface area, but with the exception of one recent paper showing increases in adolescence (Vijayakumar et al., 2016), these (Raznahan et al., 2011; Mills et al., 2014a; Wierenga et al., 2014; Ducharme et al., 2015) and the present results together support the conclusion of childhood increases followed by subtle decreases during adolescence.

After applying similar processing and analytic techniques, the results of the present multisample study showed consistent developmental patterns and trajectories for cortical structure across four longitudinal datasets with varying sample and image-acquisition characteristics. We did not observe any global increase or “peak” for cortical volume, surface area, or thickness from ages 7 to 29 in any of the four samples. The same was true for the lobar measures, except for a small early increase in frontal lobe volume in two of the samples. Our results suggest that previous inconsistencies have not primarily resulted from sample or image-acquisition differences. Rather, we speculate that they stem from the combined effects of differences in image processing, including QC procedures, and/or statistical analyses and curve fitting. All datasets in the present study were processed with an extensively used and well validated open-source software suite (Fischl, 2012) and underwent post-processing QC. Also, curve-fitting was performed with models that avoid some of the weaknesses of global polynomial models.

In both adult and developmental samples, head motion has a negative effect on estimates of cortical volume and thickness, even after excluding low-quality scans (Reuter et al., 2015; Alexander-Bloch et al., 2016). As younger participants generally move more during acquisition, motion is often confounded with age or time point (Satterthwaite et al., 2012). The importance of post-processing QC was demonstrated by Ducharme et al. (2016), who showed that exclusion of scans defined as QC failures had a large impact on identified patterns for cortical thickness development, with a shift toward more complex trajectories when scans of lower quality were included. While we attempted to limit the impact of motion by visually inspecting all reconstructed images and only included scans judged to be of adequate quality, future studies might benefit from further efforts to limit motion during data acquisition, for example by further development of on-line motion-correction procedures and quantitative motion assessment within popular software packages (Reuter et al., 2015; Greene et al., 2016; Tisdall et al., 2016). Additionally, it is likely that differences in statistical analyses may have contributed to the inconsistencies, as we recently showed that whether and how one controls for intracranial volume or total brain size influences developmental models of brain volumes (Mills et al., 2016). In relation to this, future studies are needed to investigate the consistency of reported sex differences in brain structure and development (Lenroot et al., 2007; Mutlu et al., 2013; Vijayakumar et al., 2016), using both raw and corrected measures (Marwha et al., 2017).

In addition to providing detailed descriptions of developmental patterns and trajectories of cortical structure, our results showed consistent and very strong positive relationships between cortical thickness change and volume change across nearly the entire cortex, such that relatively large reductions in thickness were associated with relatively large reductions in volume. In comparison, the relationships between surface-area change and volume change were not as strong or widespread, and for most of the occipital lobe either nonsignificant or negative. Thus, although most of the individual variation in adult cortical volume is due to variation in surface area and not thickness (Im et al., 2008), our results show that the greatest contributor to volume decrease from 7 to 29 years is thinning, as previously also shown to be the case across the adult lifespan (23–87 years; Storsve et al., 2014).

Finally, complex regional and topological patterns in the relationships between surface-area change and thickness change were observed. Across samples, both positive and negative associations were found, with positive relationships mainly seen in sulcal regions in prefrontal and temporal cortices, and negative relationships mainly seen in gyral regions in occipital cortices, paracentral cortex, and insula, and around the central sulcus. Our results mainly showed decreases with increasing age for both surface area and thickness. Thus, positive relationships indicate that relatively large reductions in surface area are associated with relatively large reductions in thickness, while negative relationships indicate that relatively large reductions in surface area are associated with relatively small reductions in thickness, and vice versa. The importance of local topology for cortical development was demonstrated in a recent large cross-sectional study finding that age-related decreases in thickness were most pronounced in the sulci (Vandekar et al., 2015), but no previous study has examined the relationships between longitudinal change in different cortical metrics on a vertex-wise level in children and adolescents (but see Storsve et al., 2014, for a study on adults, and Alemán-Gómezet al., 2013, for lobar analyses in adolescents).

The cellular and molecular changes underlying observed developmental changes in the different dimensions of the cerebral cortex and their inter-relationships remain unknown. They likely include multiple interacting processes that vary in their relative importance across regions and age (Mills and Tamnes, 2014). A recent imaging study suggests that increasing intracortical myelination is a significant driver of cortical changes in adolescence (Whitaker et al., 2016). A hypothesis for the relationships between area change and thickness change in development is that white matter growth in the form of increasing myelination and axon caliber (Yakovlev and Lecours, 1967; Benes, 1989; Benes et al., 1994) causes the cerebral cortex to stretch tangentially to the surface, expanding its area and becoming thinner, as well as improving its ability to differentiate incoming signals (Seldon, 2005, 2007). However, this does not fully explain the surface-area decrease seen in many regions in adolescence. A second hypothesis is that synaptic pruning and dendritic arborization (Bourgeois and Rakic, 1993; Huttenlocher and Dabholkar, 1997; Petanjek et al., 2011) results in decreasing gyrification and flattening of the cortex during adolescence (Raznahan et al., 2011; Alemán-Gómezet al., 2013; Klein et al., 2014) due to release of axonal tension (White et al., 2010). It is likely that a combination of these hypotheses might explain the observed complex patterns in the relationships between surface-area change and thickness change.

### Conclusion

The present results from four independent longitudinal datasets showed consistent developmental trajectories and patterns of change in cortical volume, surface area, and thickness across adolescence. Regionally variable nonlinear decreases in cortical volume and thickness, and relatively smaller steady decreases in surface area, were observed from ages 7 to 29. Further, analyses of the inter-relationships between changes in these different dimensions of the cortex revealed tight links between volume reductions and thinning, as well as regional and topological patterns in the relationships between surface-area change and thickness change.
